# Sonoelastography and Lumbopelvic Muscle Stiffness in Patients with Low Back Pain: A Systematic Review -

**DOI:** 10.31661/gmj.v12i.2465

**Published:** 2023-05-14

**Authors:** Hossein Rafsanjani Deh Qazi, Mohammad Ali Mohseni-Bandpei, Nahid Rahmani

**Affiliations:** ^1^ Department of Physiotherapy, University of Social Welfare and Rehabilitation Sciences, Tehran, Iran; ^2^ Department of Physical Therapy, School of Paramedical and Rehabilitation Sciences, Mashhad University of Medical Sciences, Mashhad, Iran.; ^3^ University Institute of Physical Therapy, Faculty of Allied Health Sciences, the University of Lahore, Lahore, Pakistan

**Keywords:** Sonoelastography, Elasticity Imaging Techniques, Muscle Stiffness, Elastic Modulus, Low Back pain

## Abstract

This study aimed to systematically review studies conducted on the application of sonoelastography (SE) to evaluate lumbopelvic muscle stiffness in patients with low back pain (LBP). All relevant articles were retrieved from the available electronic databases, including PubMed, Web of Science, Scopus, EMBASE, Cochrane library, and CINAHL, using the keywords “Sonoelastography”, “Elasticity Imaging Technique”, “Muscle Stiffness”, “Modulus Elasticity”, “Low Back Pain”. After initial searches, studies that met the inclusion criteria (i.e., published in English and sonoelastography were used to assess lumbopelvic muscle stiffness in both patients with LBP and healthy individuals) were enrolled. Also, any animal research, abstract of the seminar and/or conference, and/or non-English-language article were excluded. The quality of the studies was assessed using the Physiotherapy Evidence Database (PEDro) scale. In total, eight relevant studies were selected for review. Three studies were considered to have excellent quality, and five were considered fair quality using the PEDro scale. All reviewed studies have reported that SE can be considered a non-invasive method for quantifying changes in lumbopelvic muscle stiffness. Muscle stiffness was significantly higher in LBP patients compared to healthy persons, as well as across subgroups of LBP patients in various test postures (P˂0.05). Only one study was conducted on the reliability of SE in healthy individuals, while another examined the validity of SE imaging. The results of the present systematic review indicated that SE imaging is a reliable and valid tool to identify muscle changes that occur in patients with LBP and evaluate the effects of rehabilitation treatment.

## Introduction

As one of the most important challenges for the healthcare system, low back pain (LBP) is considered one of the most commonly referred reasons to medical centers worldwide [1]. According to reports, it is the sixth most prevalent cause of medical consultations in the United States [2]. According to data from other nations, including France, LBP has been widespread and has had economic and social consequences [3]. In Iran, reports suggest that the lifetime prevalence of LBP among nurses and pregnant women is 62% and 84%, respectively [4][5]. Additionally, 33.7% of work absenteeism was reported by nurses within one month [5]. Muscle changes in patients with LBP were identified in posterior trunk muscles, including the erector spine [6] and lumbar multifidus [7], which are reported to play an important role in spinal dynamics [8].Moreover, these changes may occur in the abdominal muscles, including the internal oblique and, in particular, transverse abdomens. These muscles are renowned for giving the spine lateral and rotational control, as well as for transmitting stress to the thoracolumbar fascia and assisting in controlling intra-abdominal pressure levels [9]. Muscle atrophy and increased fat volume of muscle tissue affect its function [10] as well as physical performance [11]. Several studies have identified that ipsilateral muscle atrophy of the lumbar multifidus has been significant in patients with unilateral LBP compared to healthy subjects [12][13].Various imaging techniques, such as ultrasound, computed tomography scan, and magnetic resonance imaging, are available to assess muscle shape, size, and stiffness [14]. Ultrasound is considered one of the most accessible, inexpensive, and reliable imaging equipment without ionizing waves compared to other imaging techniques [15].Recently, sonoelastography (SE) as a non-invasive high-resolution resolute method to quantify tissue stiffness has also been reported to detect the probable changes in muscle tissue through two primary techniques, namely, strain elastography (SE) and shear wave Sonoelastography (SWE) [16]. While the former technique visualizes tissue deformation with compression applied by the examiner, shear waves are produced in the latter by a transducer, which calculated Young’s elastic modulus [17]. It might, then, give accurate stiffness values in selected areas inside the measurement box. Considering the role of core muscles stiffness in the stability of the spine, and SE as a valuable modality to characterize mechanical properties of muscles and mechanical heterogeneity index, this study aimed to review validity and reliability of SE in evaluating the mechanical characteristics of lumbopelvic muscles in both healthy participants and patients with LBP.

## Materials and Methods

*Search Strategy*

All related articles were found through electronic search in the available databases, including PubMed, Web of Science, Scopus, EMBASE, Cochrane library, and CINAHL, using the following key terms until March 2022: "Sonoelastography", "Elasticity Imaging Technique," "Muscle Stiffness," "Modulus Elasticity," and "Low Back Pain." based on MeSH terms strategy as: (muscle stiffness; OR muscle; OR stiffness; OR low back pain; OR back pain; OR modulus elasticity; OR strain ratio; OR elasticity ratio) AND (sonoelastography; OR real time elastography; OR sonoelastography; OR elastography: OR elasticity imaging technique). The search was completed by reviewing the reference lists at the end of all related articles. 

*Selection of Studies*

To select the eligible articles based on inclusion/exclusion criteria, two authors (NR and HR) independently reviewed the titles and abstracts after completing the initial electronic search. The studies evaluated if SWE imaging of lumbopelvic in patients with LBP and healthy subjects were investigated. All relevant articles included the application of SE imaging to assess the lumbopelvic muscles stiffness (i.e., multifidus, quadratus lumborum, gluteus medius, piriformis) in both normal individuals and patients with LBP and also, published in the peer-reviewed journals in the English language. Hence, any studies that used animals or assessed muscles other than the lumbopelvic muscles, presentations at a seminar and/or conference, and non-English articles were excluded. The two authors’ agreement allowed for the selection of the research to be made in the end.

*Data Extraction and Analysis*

At this stage, the two authors (NR and HR) individually extracted the necessary data from the entered studies. The two authors reviewed each of the eight studies (NR and HR). The information extracted regarding the methods was as follows: study design, study participants, description of SE technique, description of intervention for different treatments, the participants’ position, control groups, and measurement of study variables. The SE imaging method was found to be fair to excellently reliable based on Intra Correlation Coefficients (ICCs) ranging from 0.44 to 0.92, respectively [17]. The Research Ethics Committee of University of Social Welfare and Rehabilitation Sciences approved the study (approval number: IR.USWR.REC.1399.059).

## Results

*Selection of Studies and Their Characteristics*

**Figure-1:**
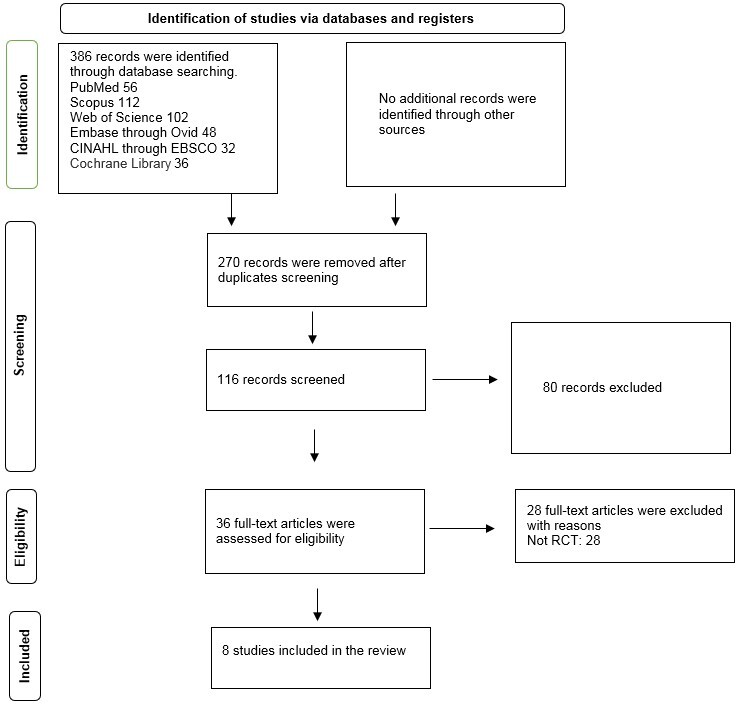
Figure 1- PRISMA Flow diagram of the study

The electronic search yielded 386 records, and after duplication screening, 116 records remained. Based on the inclusion/exclusion criteria, 80 studies were excluded by reading the titles and abstracts, and only 36 articles were eligible for the assessment. The authors studied the full text of 36 articles, of which 28 were excluded based on exclusion criteria, and eight articles with 407 participants were included in the main analysis. The PRISMA flow diagram is presented in Figure-1.

Among eight studies, six [18][19][20][21][22][23] were conducted on monitoring rehabilitation programs such as stabilization, manipulation, and general exercise. Whereas one study [24] [25] has exclusively considered the reliability of SE imaging in normal individuals, another study [25]investigated the validity of SE imaging.

*Quality Appraisal*

Two authors (NR and HR) who performed baseline data searches assessed the methodological quality of the identified using the PEDro scale. The total PEDro scores of 0-3 are considered poor, 4-6 as fair, and 7-10 as excellent [26]. While three of the studies [18][20][22] were reported to have an excellent quality status (PEDro score˃7), five studies [19][21][23][24][25] were determined as fair quality studies (PEDro scores: 4 and 6). All eight studies met four PEDro requirements (random grouping, application of the same qualitative study factors at the start of the study among groups, measurement of at least one common output variable in 85% of participants, and comparison of at least one fundamental study variable in the two groups). In none of the studies were the researchers blinded to evaluate variables. Table-1 contains the PEDro scores for each study.

**Table:** Table 1. Physiotherapy Evidence Database (PEDro) Scoring of Included Studies

**Study**	**Neto *et al*. [18]**	**Koppenhaver *et al*. [19]**	**Chan *et al*. [20] **	**Gao *et al*. [21]**	**Masaki *et al*. [22]**	**Murillo *et al*. [23]**	**Koppenhaver *et al*. [24]**	**Tier *et al.* [25]**
**2 **	Y	Y	Y	Y	Y	Y	N	N
**3**	Y	Y	N	N	N	N	N	N
**4 **	Y	Y	Y	Y	Y	Y	N	N
**5**	Y	Y	Y	N	N	Y	N	N
**6 **	N	N	N	N	N	N	N	N
**7**	Y	N	N	N	Y	N	N	Y
**8**	Y	Y	Y	Y	Y	Y	Y	Y
**9**	Y	Y	Y	N	N	N	Y	Y
**10**	Y	Y	Y	Y	Y	Y	Y	Y
**11**	Y	Y	Y	Y	N	Y	Y	N
**Total Score**	9/10	8/10	7/10	6/10	5/10	6/10	4/10	4/10
**Quality**	Excellent	Excellent	Excellent	Fair	Fair	Fair	Fair	Fair


**Y:** Criterion satisfied; **N:**Criterion not satisfied **2.** Random allocation to group; **3. **Allocation was concealed; **4.** Similar groups aft baseline regarding prognostic factors; **5.** Blinding of all subjects; **6.** Blinding of therapist who administered the therapy;**7.** Blinding of all assessors who measured at least one key outcome; **8.** Measure at least one outcome for more than 85% of subjects; **9.** All subjects who received the intervention or “intention to treat” were stated; **10.** Between-group statistical comparison for at least one key outcome; **11.** Point measures and measures of variability for at least one key outcome.

**Table:** Table 2. Details of Trials Evaluating Lumbopelvic Muscles Stiffness Using Sonoelastography in Patients with Low Back Pain (LBP) and Healthy Subjects.

**Study**	**Purpose of study**	**Participants**	**Intervention**	**Outcome measure**	**Participants position**	**Results**	**Study design**
**Neto *et al.* [18]**	To evaluate, elasticity, cross-section area of the multifidus for the contractile function.	The study included 12 adults male with chronic LBP and 12 asymptomatic male controls.	The patients were in prone, upright, and 25^° ^and 45^°^ forward stooping positions.	The elasticity of the multifidus at the L4 level.	Prone position	There was an increasing stiffness of multifidus from the prone to upright position and 25^° ^and 45^°^ forward stooping positions. Differences in multifidus stiffness between chronic LBP and control group were shown in the upright and 25^° ^and 45^°^ forward stooping positions but not in the prone position.	Randomized control trials
**Koppenhaver *et al.* [19]**	To evaluate the association of LBP with muscle stiffness and muscle mass of the lumbar back muscle in young and middle-aged medical workers.	The study included 9 medical workers with LBP and 23 asymptomatic medical workers (control group).	The patients were in prone position.	Muscle stiffness and mass of the lumbar back muscle (lumbar erector spine, quadratus lumborum, multifidus).	Prone position	There was significantly higher lumbar multifidus stiffness in the LBP group than that in the control group.	Randomized control trials
**Chan *et al.* [20]**	To evaluate lumbar back muscle stiffness in people with chronic LBP- related leg pain.	The study included 8 patients with unilateral LBP- related leg pain and 8 healthy controls.	The subjects received passive ankle dorsi flexion performed at 2^°^/s in an isokinetic dynamometer.	Lumbar back muscle and sciatic nerve stiffness.	Sitting position	In people with LBP - related leg pain, the affected limb showed higher muscle and sciatic nerve stiffness compared to unaffected limb.	Randomized control trials
Gao *et al*. [21]	To evaluate differences in passive muscular stiffness between the superficial multifidus and deep multifidus and to compare their passive and active stiffness in individuals with LBP and asymptomatic subjects.	The study included 15 LBP individuals and 15 asymptomatic individuals (control group).	The patients were in prone position for 5 minutes to measure passive muscular stiffness and to measure active muscular stiffness patients were acquired during an isometric trunk extension.	Active and passive lumbar muscle stiffness.	Prone position	Deep multifidus displayed higher passive muscular stiffness than superficial multifidus in both the control and LBP groups. Patients with LBP showed higher passive muscular stiffness of superficial multifidus and lower contraction ratio compared to control group.	Randomized control trials
Masaki *et al*. [22]	To evaluate resting and contracted stiffness of lumbar muscle in individuals with and without LBP.	The study included 60 individuals with LBP and 60 asymptomatic individuals (control group).	The lumbar erector spine was imaged at rest only, while the lumbar multifidus was imaged at rest and during contraction.	Lumbar muscle shear modulus.	Prone position	Stiffness of the erector spine and lumbar multifidus at rest (but not during contraction) was greater in participants with LBP than in asymptomatic controls.	Randomized control trials
Murillo *et al.* [23]	To evaluate the application of ultrasound shear wave elastography in assessing lumbar muscle changes after OPM	The study included 20 patients with LBP and 9 aged match volunteers.	The shear wave was measured in muscle relaxation and contraction in all participants and immediately before and after OPM in patients.	Shear wave elastography in iliocostalis lumborum muscle.	Prone position	The iliocostalis muscle shear wave elastography significantly differed between patients with LBP and healthy volunteers, between muscle relaxation and contraction, and before and after OPM.	Randomized control trials
Koppenhaver *et al.* [24]	To assess intra - rater and test - retest reliability of shear wave elastography elasticity measures of the lumbar erector spine and multifidus muscles during rest and differing levels of contraction in asymptomatic individuals.	The study included 36 healthy volunteers.	This single - group repeated - measures design involved a base line measurement session and a follow up session 3 days later. The lumbar multifidus was imaged at rest and during three levels of contraction (minimal, moderate, and maximally). The lumbar erector spine (iliocostalis and longissimus muscles) were imaged at rest only.	Intra - rater and test - retest reliability of shear wave elastography.	Prone position	Overall reliability estimates were fair to excellent with ICCs ranging from 0.44 to 0.92.Reliability was higher in the lumbar multifidus muscles than the erector spine muscles, slightly higher during contraction than during rest, and substantially improved by using the mean of 3 measurements.	Reliability Study
Tier *et al.* [25]	To evaluate relationship between shear modulus and myoelectric activity of lumbar multifidus and longissimus muscles to assess its validity.	The study included 9 healthy participants.	Participants performed isometric trunk extension in side - lying from 0 to 30% maximal volunteers contraction with (EMG) amplitude feedback	Shear wave elastography and intramuscular electromyography of multifidus at L4/5, longissimus at L2, were recorded.	Side lying	Generally, shear modulus was moderately correlated with RMS EMG (0.50-.078).Although a linear relationship between shear modulus/EMG was confirmed, supporting validity of shear wave elastography measures in anatomically distinct back muscles, this depends on image quality.	Validity Study

**LBP: **Low back pain; **OPM: **Osteopathic manipulative treatment;** ICC: **Intraclass correlation coefficients; **RMS: **Root mean squared; **EMG: **Electromyography.

## Discussion

Despite the high prevalence of LBP among adult populations, no specific imaging modality has so far been proposed as gold standard. Sonoelastography has been reported in animal models as the proper imaging technique to define the degree of stiffness in lumbopelvic muscles. In this systematic review study, for the first time, we evaluated the SE in adult patients with LBP. Our findings showed that SE can be a potential instrument for defining the extent of stiffness in adult patients with LBP. (Table-2) In this systematic review, three out of eight relevant studies were considered excellent and five were fair quality based on PEDro scale. According to previous evidence, SE may be used as a non-invasive approach to measuring the stiffness changes in lumbopelvic muscles [26]. Detectable variations in muscular stiffness were found between LBP patients and healthy persons or between various subgroups of LBP patients [27][28]. Six out of the eight aforementioned studies monitored rehabilitation programs including stabilization, manipulation, and general exercise. Muscle stiffness has recently been evaluated in many research. As stated in the aforementioned six studies, muscle stiffness in lumbopelvic sonoelastography decreases in patients with LBP after rehabilitation. 

Using SE imaging, Chan *et al*. [20] investigated how various lumbar postures affected the flexibility of the lumbar multifidus.By increasing the effectiveness of Young’s modulus from the prone to the upright position, a growing multifidus stiffness was demonstrated [29]. Significant alterations in the superficial and deep multifidus muscles were found in the data, indicating that there had been changes in the muscles’ stiffness during both rest and exercise [30]. Koppenhaver *et al*. [19] also showed that the stiffness in superficial muscles (multifidus, etc.) is lower than deeper ones (quadratus lumborum) after rehabilitation. Another study used SE to compare the lumbar spine muscles’ relaxed and contracted stiffness in people with and without LBP [31]. Individuals with LBP were shown to have higher levels of resting lumbar muscle stiffness than asymptomatic controls, and this stiffness was linked to self-reported pain and disability. 

Pathological and morphological changes following LBP occur in lumbopelvic muscles cannot be treated simultaneously [32]. In a different study, Masaki *et al*. [22] looked at the connection between LBP and muscle mass and stiffness in young and middle-aged nurses. In comparison to the control group, the lumbar multifidus stiffness in the LBP group was considerably higher. Other studies have examined the stiffness of the lumbar back muscles in people who had chronic leg pain brought on by LBP. According to the findings, patients with LBP-related leg discomfort had stiffer muscles and sciatic nerves in the affected limb than in the unaffected limb. Another study assessed the use of SE in evaluating lumbar muscle alterations following osteopathic manipulative treatment (OPM) [23]. The iliocostalis lumborum muscle SE significantly differed (OMT) between patients with LBP and healthy volunteers, between muscular tension and relaxation, and between before and after osteopathic manual treatment [33].Koppenhaver *et al*. [24] evaluated the intra-rater and test-retest reliability of sonoelastographic elasticity measures of erector spine and multifidus muscles during rest and different contraction levels in asymptomatic individuals (n=30). The overall reliability was estimated as fair to excellent with ICCs ranging from 0.44 to 0.92 [34].Their results suggested sonoelastography as a reliable method for lumbopelvic muscle stiffness assessment in healthy individuals and patients with LBP.According to Tier *et al*., [25] the lumbar muscle shear modulus is moderately correlated with the root mean square of EMG, which was in agreement with the previously confirmed linear relationship between the shear modulus and EMG activity of muscles.These results suggest that sonoelastography is a reliable and valid tool to assess the elasticity index of lumbopelvic muscles in patients with LBP and healthy individuals.While methodological flaws were found in some studies, their small sample sizes, lack of reliable sonoelastography imaging parameters, and lack of a common definition for LBP are considered as the most important limitations of the study [35].

## Conclusion

According to the results of this review, SWE can be used for clinical evaluation of the effect of rehabilitation programs in patients with LBP. The strengths of this review study include a strong electronic search strategy, identification of a framework for robust review methodology, and the quality of assessment of the researched variables. Sonoelastography imaging is a useful, reliable, and valid method in evaluating lumbar muscle stiffness. 

## Conflict of Interest 

The authors declare that they have no competing interest.
